# The clinicopathological and molecular features of sporadic gastric foveolar type neoplasia

**DOI:** 10.1007/s00428-020-02846-0

**Published:** 2020-06-12

**Authors:** Tamotsu Sugai, Noriyuki Uesugi, Wataru Habano, Ryo Sugimoto, Makoto Eizuka, Yasuko Fujita, Mitsumasa Osakabe, Yosuke Toya, Hiromu Suzuki, Takayuki Matsumoto

**Affiliations:** 1 Department of Molecular Diagnostic Pathology, School of Medicine, Iwate Medical University, 2-1-1, Shiwagun, Yahabachou, 028-3695 Japan; 2 Department of Pharmacodynamics and Molecular Genetics, School of Pharmacy, Iwate Medical University, 2-1-1, Shiwagun, Yahabachou, 028-3695 Japan; 3 Division of Gastroenterology, Department of Internal Medicine, School of Medicine, Iwate Medical University, 2-1-1, Shiwagun, Yahabachou, 028-3695 Japan; 4 Department of Molecular Biology, School of Medicine, Sapporo Medical University, Sapporo, Japan

**Keywords:** Allelic imbalance, *APC* promoter 1B, DNA methylation, Foveolar type neoplasia, Gastric intraepithelial neoplasia

## Abstract

Gastric intraepithelial foveolar type neoplasia (IEFN) is not well defined. In addition, atrophic mucosa (AM) is an important issue to consider when evaluating gastric tumorigenesis. Here, we assessed the clinicopathological characteristics and molecular alterations contributing to the development of IEFN compared with intestinal type neoplasia. We examined the clinicopathological and molecular features of 42 cases of IEFN with low-grade dysplasia (LGD) and those of 77 cases of intraepithelial intestinal type neoplasia (IEIN) with LGD. The clinicopathological and molecular features examined included the AM status, mucin phenotype expression, CDX2 expression, p53 overexpression, β-catenin intranuclear accumulation, microsatellite instability (MSI), DNA methylation status (low methylation epigenotype [LME], intermediate ME, or high ME), allelic imbalances (AIs), and *APC* promoter 1B mutations. There were no differences in the frequencies of AM and rates of CDX2 expression between IEFN and IEIN cases. Although no differences in the frequencies of p53 overexpression and MSI were observed between the two histological types, intranuclear expression of β-catenin was significantly higher in IEIN than in IEFN. In addition, although the rate of LME was significantly higher in IEFN cases than in IEIN cases, IEFN was characterized by AIs at multiple foci. Finally, mutation of the *APC* promoter 1B, which is a characteristic of gastric adenocarcinoma and proximal polyposis of the stomach (potentially resembling IEFN), was detected in only one IEFN case. These findings suggested that IEFN may be an independent entity in terms of molecular alterations including the presence of multiple AIs and LME.

**Electronic supplementary material:**

The online version of this article (10.1007/s00428-020-02846-0) contains supplementary material, which is available to authorized users.

## Introduction

Gastric cancer (GC) is one of the most common cancers worldwide [1]. GC is a heterogeneous disease with various histological patterns, some of which have been demonstrated as independent clinicopathological entities [2]. Such histological types associated with one of the prognostic factors are described in the World Health Organization (WHO) classification of GCs [2]. Gastric differentiated type intraepithelial neoplasia is largely classified into intestinal and gastric types, including foveolar type and pyloric type [2]. In gastric type neoplasia, foveolar type neoplasia (FN), which is also described as a foveolar adenoma/dysplasia, is a rare histological entity of GC [2–5]. Although this type was described in a recently published WHO report, the histological criteria for evaluation of FN are not well defined [2–5]. According to the WHO classification, gastric differentiated type intraepithelial neoplasia is divided into low (LGD)- and high-grade dysplasia (HGD) [6]; this classification may also apply to intraepithelial FN (IEFN). The presence of LGD can make it difficult to differentiate neoplastic from non-neoplastic (e.g., hyperplasia) tumors by pathologists [7]. To resolve these issues, detailed molecular examination is needed.

According to the genomic classification of The Cancer Genome Atlas, GC can be divided into four subgroups: (1) tumors positive for Epstein–Barr viral infection, (2) those with microsatellite instability (MSI)—high, (3) those with genomic stability, and (4) those with chromosomal instability [8]. This classification is made based on genetic alterations, epigenetic alterations, and abnormalities in cancer-related proteins. It is widely accepted that the accumulation of various genetic and epigenetic alterations in normal cells can induce their transformation into neoplastic or malignant cells [8]. Genetic alterations include allelic imbalance (AI), regarded as loss of heterozygosity, copy number alterations, and genetic mutations [8–12]. AI and copy number alterations may promote malignant transformation of tumor cells [10]. In addition, MSI caused by mismatch repair deficiency also plays a major role in a subset of GCs [13, 14], while epigenetic alterations have been demonstrated to be responsible for tumor development [8, 10, 13]. Due to the critical role of epigenetic alterations during tumor progression, epigenetic characterization of tumor cells might help with understanding their progression [13].

Various markers closely associated with gastric carcinogenesis have been examined in GC cases [9–11, 15]. These markers include intranuclear accumulation of β-catenin (associated with disruption of Wnt signaling), cellular phenotype (intestinal versus gastric phenotype), CDX2 expression, cellular proliferation, and p53 overexpression/mutations [9, 10, 16]. Therefore, it will be important to identify differences in genetic alterations between IEFN and intraepithelial intestinal type neoplasia (IEIN).

To further our understanding of this putatively novel subtype of GC, we examined the clinical, pathologic, immunohistochemical, and molecular features of gastric FN/D cases.

## Materials and methods

### Patients

**Fig. 1:**
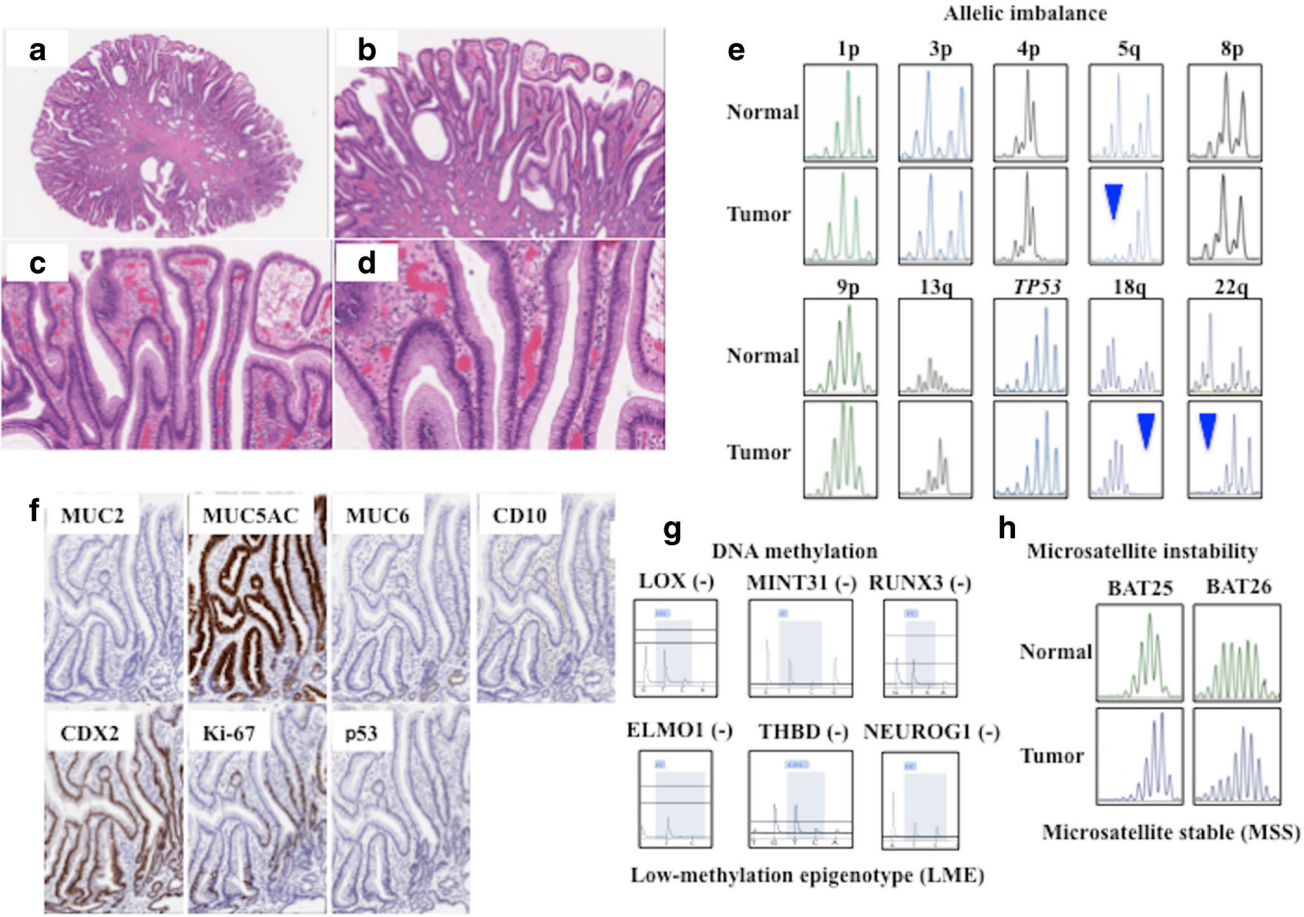
Representative findings in intraepithelial foveolar type neoplasia. **a** Histological images. **b** Low magnification. A papillary structure is seen. **c** Medium magnification. Columnar epithelial cells with small-sized nuclei are seen. **d** High magnification. Nuclei are seen in the basal layer. **e** Allelic imbalances observed at multiple foci (5q, 18q, and 22q). Note arrow head. **f** Immunohistochemical staining of the indicated markers. Positive expression of MUC5AC and CDX2 is found. **g** DNA methylation analysis indicating a low methylation status. **h** Microsatellite analysis indicating microsatellite stability

**Fig. 2:**
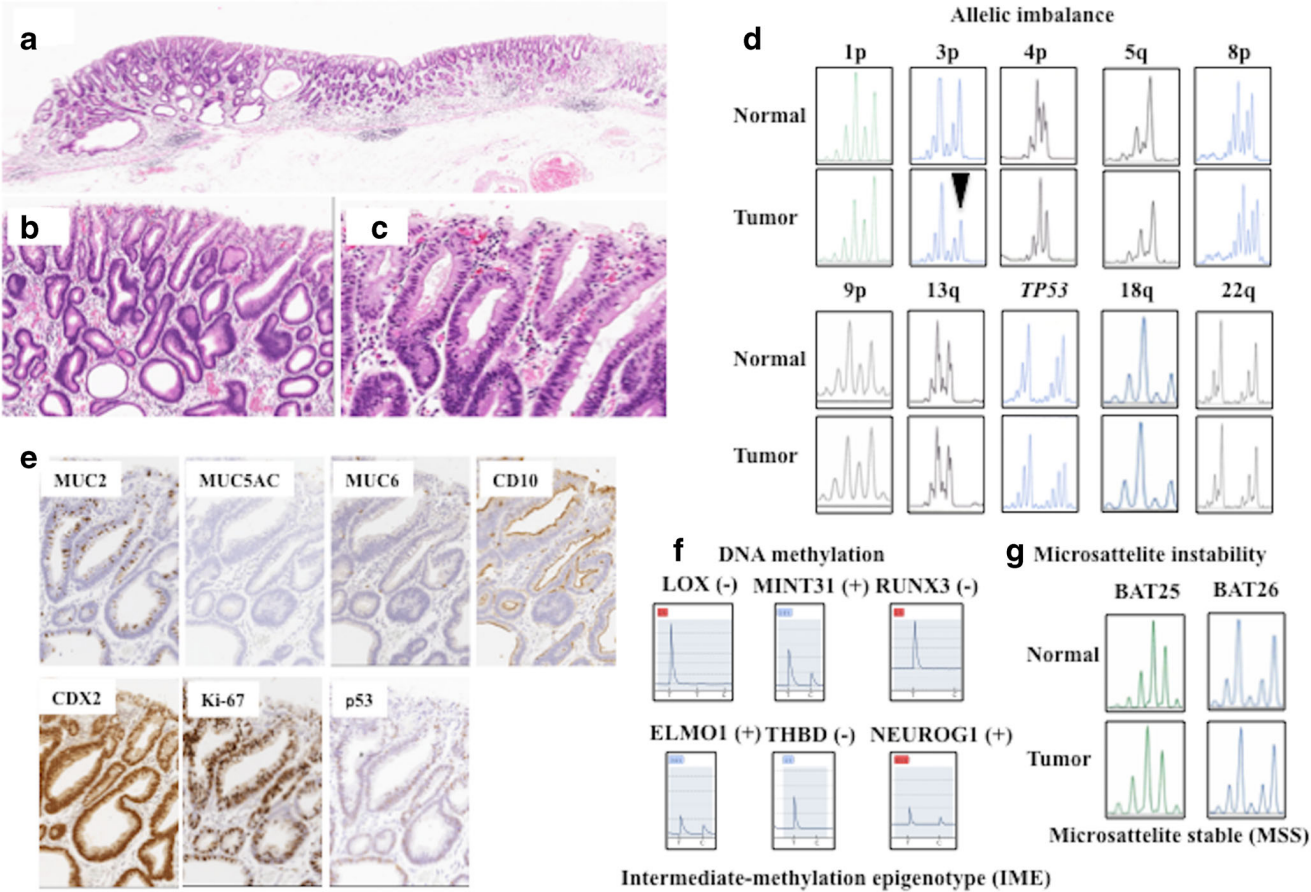
Representative findings in intraepithelial intestinal type neoplasia (low-grade dysplasia). **a** Histological images. **b** Low magnification. A tubular structure is seen. **c** High magnification. Columnar epithelial cells with intermediate-sized nuclei are present. **d** Allelic imbalances observed at two foci (3p). Note arrow head. **e** Immunohistochemical staining of the indicated markers, showing positive expression of MUC2, CD10, and CDX2. No expression of MUC5AC and MUC6. No overexpression of p53. **g** DNA methylation analysis indicating intermediate methylation status. **h** Microsatellite analysis indicating microsatellite stability

The study included 42 patients with gastric IEFN diagnosed at Iwate Medical University Hospital and its related hospitals during 2015–2019. In addition, 77 patients with gastric IEIN were included and compared with patients with IEFN. All tumors were removed by endoscopic resection. Approximately 10 slides containing primary tumor specimens from each patient were prepared for hematoxylin and eosin (HE) and immunohistochemical staining. Primary histopathology reports were available for all patients, and the age, sex, lymph node status, vascular invasion status, differentiation type, and tumor invasion depth of each patient were recorded. These clinicopathological findings were assessed according to the general rules for the management of GC established by the Japanese Gastric Cancer Association [17]. Briefly, histologically, foveolar IEFN shows cuboidal to columnar cells with pale-to-clear cytoplasm and hyperchromatic round-to-oval nuclei (low nucleus to cytoplasm ratio [N/C]). Foveolar-like cells with irregular glandular branching and epithelial folding are also frequently noted in the foveolar type, whereas goblet and Paneth cells are rarely identified. In addition, papillary or villous surface structures are frequently found in this type. To confirm the histological diagnosis of IEFN, immunohistochemically positive expression of MUC5AC was assessed. Conversely, intraepithelial intestinal type neoplasia (IEIN) resembles colonic adenoma and is composed of large to moderate tubules lined by basophilic columnar cells with hyperchromatic pencillate nuclei with a slight pseudostratification and low N/C ratio. Goblet and Paneth cells are commonly observed in IEIN. The “hybrid type” proposed by Park et al. was not found in the current study [4]. In addition, mucosal atrophy and intestinal metaplasia were examined in the surrounding mucosa of the IEFN and IEIN cases. The clinicopathological characteristics of the IEFN and IEIN patients are shown in Table 1. Two experienced pathologists (T.S. and N.U.) determined the diagnosis of each case examined by consensus. The representative histological features of the IEFN and IEIN cases are shown in Figs. 1 and 2.

**Table 1:** Clinicopathological findings of intraepithelial foveolar type neoplasia and intraepithelial intestinal type neoplasia

		IEFN (%)	IEIN (%)	*p* value
Total		42	77	
Sex	Man:woman	28:14	58:19	0.3135
Age (year)	Range (median)	25–87 (71)	54–87 (72)	0.5203
Size (mm)	Range (median)	4–53 (15)	10–103 (19)	0.3372
Locus	Upper	8 (19.0)	14 (18.1)	0.4108
	Middle	13 (31.0)	33 (42.9)	
	Lower	21 (50.0)	30 (39.0)	
Macroscopic type	Protruded type	7 (16.7)	2 (2.6)	0.0050
	Flat elevated type	27 (64.3)	40 (51.9)	
	Flat type	1 (2.4)	7 (9.1)	
	Depressed type	7 (16.7)	28 (36.4)	
Mucosal atrophy	Negative	0 (0)	0 (0)	N.S.
	Positive	42 (100)	77 (100)	
Intestinal metaplasia	Negative	0 (0)	1 (1.3)	0.4583
	Positive	42 (100)	76 (98.7)	

*IEFN*, intraepithelial foveolar type neoplasia; *IEIN*, intraepithelial intestinal type neoplasia; *N.S.*, not significant

Informed consent was obtained from all patients, and our study was approved by the ethics committee of Iwate Medical University (reference number: MH2018-009).

### Immunohistochemical analysis

Sections of formalin-fixed, paraffin-embedded tissue blocks were cut at a 3–4-μm thickness for immunohistochemical analysis using an extensive panel of antibodies, including anti-p53 (DO7; DAKO, Copenhagen, Denmark), anti-MUC2 (Ccp58; Novocastra Laboratories, Newcastle, UK), anti-MUC5AC (CLH2; Novocastra Laboratories), anti-MUC6 (CLH5; Novocastra Laboratories), anti-CD10 (56C6; Novocastra Laboratories), anti-caudal-related homeobox transcription factor 2 (CDX2; DAK-CDX2, ready to use; Agilent Technologies), anti-β-catenin (clone 14; Becton Dickinson), and anti-Ki-67 (MIB1, monoclonal; DAKO) antibodies. The sections were prepared, dried, deparaffinized, and rehydrated before subjecting to microwave treatment (H2500, Microwave Processor; Bio-Rad Laboratories, Hercules, CA, USA) in citrate buffer (pH 6.0) for 5 min. The slides were counterstained with hematoxylin, dehydrated, and then mounted. Immunohistochemical staining was examined using the Envision+ System (DAKO).

### Assessment of immunohistochemical expression

In order to avoid arbitrary evaluation, we used the following criteria to analyze immunohistochemical staining of mucin markers (MIUC2, MUC5AC, and MUC6), CD10, β-catenin, CDX2, and p53. The staining intensity scores were divided into four categories: no staining, weak/equivocal staining, moderate staining, and strong staining. Moderate or strong staining was considered as positive expression. The percentage of cells with positive expression was scored as follows: 0, 0–10% cells; 1, 10% to < 30% cells; 2, 30% to < 60% cells; 3, 60% to < 100% cells; and 4, 100% cells. In this study, a score of greater than 1 was classified as positive expression of the markers in the lesions, based on the finding that the inflection point on the histogram for the markers examined was greater than 1 (a useful method to objectively set the cut-off value; Supplementary Figures 1 and 2).

### Phenotype classification

Immunopositivity in greater than 10% and less than 10% of tumor cells (scores of 0 and 1 versus scores of 2 and 3) was regarded as positive and negative expression, respectively (Supplementary Figure 1-a–d). In the current study, the gastric tumors were classified into four groups according to their immunostaining pattern. The gastric phenotype was defined by positive expression of the gastric mucin MUC5AC and/or the pyloric gland mucin MUC6 but negative expression of MUC2. The intestinal phenotype was defined by positive expression of MUC2 and/or CD10 (along the brush border). Intestinal type tumors were subclassified into two groups: large intestinal (positive for MUC2 only) and small intestinal phenotype (positive for CD10 only). Mixed type tumors were defined by an immunostaining pattern consistent with both the gastric (positive expression of MUC5AC and/or pyloric gland mucin) and intestinal (positive expression of CD10 and/or MUC2) phenotypes. Finally, tumors that were not classified as the gastric or intestinal phenotype were assigned to the “unclassified” phenotype.

### CDX2 expression

For CDX2, nuclear staining of these markers was considered positive expression. For CDX2 expression, immunopositivity in greater than 10% (scores of 2–4) versus less than 10% (scores of 0 or 1) of tumor cells was also used based on the criterion for defining positive versus negative expression, respectively (Supplementary Figure 2-a).

### p53 overexpression

According to the criteria, the cut-off value for p53 overexpression in the study was determined to be greater than 10% (> score 2) according to Supplementary Figure 2-b.

### β-Catenin immunostaining

Immunostaining of β-catenin in the nucleus was considered positive and in the membranes as negative. β-Catenin-positive cells greater than 10% (> score 2) was classified as positive (Supplementary Figure 2-c).

### DNA extraction

Microdissection of formalin-fixed, paraffin-embedded tumor and non-tumor mucosal sections was performed on hematoxylin-stained slides. The tumor and non-tumor mucosal components were microdissected separately and incubated in 50 μL buffer (0.5% Tween-20 [Boehringer Mannheim, Ingelheim, Germany], 20 μg proteinase K [Boehringer Mannheim], 50 mM Trizma base, pH 8.9, and 2 mM ethylenediaminetetraacetic acid) at 56 °C for 12–18 h. Proteinase K was inactivated by incubating the samples at 100 °C for 10 min. All tumor samples in which the neoplastic cells accounted for at least 50% of the cell population were evaluated.

### Analysis of MSI

MSI analysis was performed as described previously. Five different microsatellite loci, BAT25, BAT26, D5S346, D2S123, and D17S250, recommended by the Bethesda panel for evaluation of MSI in colon cancer, were assessed in this analysis [18]. A tumor was defined as positive for MSI when polymerase chain reaction (PCR) resulted in an abnormal DNA band size compared with the corresponding non-cancer sample for the multiple loci evaluated. MSI-positive colorectal carcinomas were used as controls in this study and were divided into two groups, those with high-level instability (MSI at ≥ 40% of loci) and those with low-level instability (MSI at < 40% of loci), as described previously [18]. Tumors with an alteration in only one marker and those categorized as having low-level instability were considered to be microsatellite stable in this study.

### DNA methylation analysis

DNA methylation at the promoter regions of six genes, originally proposed by Yagi et al., was quantified using the PyroMark Q24 system (QIAGEN, Hilden, Germany) [19, 20]. The cut-off value of methylation status was determined to be 15%. Tumors with methylation of at least two of three markers (*RUNX3*, *MINT31*, and *LOX*) were defined as having a highly methylated epigenotype (HME). The remaining tumors without HME were screened for methylation of three other markers (*NEUROG1*, *ELMO1*, and *THBD*) and were defined as having the intermediate methylation epigenotype (IME) if at least two of these markers were methylated. Tumors not classified as HME or IME were defined as having the low methylation epigenotype (LME).

### PCR analysis of AI

AIs at 1p, 3p, 4p, 5q, 8p, 9p, 13q, *TP53*, 18q, and 22q chromosomal regions were examined in paired tumor and normal tissues obtained from 107 patients (42 IEFN and 65 IEIN cases) using 22 highly pleomorphic microsatellite markers (D1S228, D1S548, D3S2402, D3S1234, D4S2639, D4S1601, D5S107, D5S346, D5S299, D5S82, D8S201, D8S513, D8S532, D9S171, D9S1118, D13S162, *TP53*, D18S487, D18S34, D22S274, D22S1140, and D22S1168). AIs at these microsatellite markers have been reported frequently in GC [10]. Microsatellite sequences were amplified by PCR using specific primers, obtained from the Genome Database (http://gdbwww.gdb.org/gdb/), and a thermal cycler (GeneAmp PCR System 9600; PerkinElmer, CA, USA), as described previously [20]. If the expression of at least one of the plural markers examined within a chromosomal locus was classified as positive, the AI status of that locus was considered positive.

The peaks produced by PCR for a microsatellite marker in the normal tissue DNA samples were used to determine whether the tumor sample was homozygous (one peak) or heterozygous (two peaks) for that microsatellite marker. The allelic ratio was calculated as described by Habano et al. [21]. A tumor was considered to have AI if the allele ratio was less than or equal to 0.60.

### Analysis of mutations in *APC* promoter 1B

Mutations in *APC* promoter 1B, which is a mutational hotspot in gastric adenocarcinoma and proximal polyposis of the stomach, were examined by single-strand conformation polymorphism analysis and then confirmed by sequencing analysis. Single-strand conformation polymorphism analysis was performed as described previously [22], with some modifications. Briefly, *APC* promoter 1B was amplified by PCR, and the PCR products (2 μL) were mixed with 10-μL gel loading solution (9.5% deionized formamide, 20 mM EDTA–Na, 0.05% xylene cyanol and bromophenol blue), denatured at 95 °C for 5 min, and kept on ice until loading onto the gel. A non-denaturing 7.5% polyacrylamide gel was used for electrophoresis, which was performed at 260–300 V at 22 °C for 3–12 h using a temperature controller (Resolmax; ATTO Co., Tokyo, Japan). The gels were visualized by silver staining and photographed. Direct sequencing of the PCR products was performed as described previously [22]. Finally, the primer sequences used for nested PCR are listed in Supplementary Table.

### Statistical analysis

Differences in histological features, immunohistochemical findings, and the MSI, methylation, and AI statuses were analyzed by the chi-square test using StatMate III (Atom, Tokyo, Japan). Differences in age distribution among the two groups were evaluated by the Kruskal–Wallis *H* test using StatMate III. Differences with *p* values of less than 0.05 were considered significant.

## Results

### Differences in the clinicopathological characteristics of the IEFN and IEIN cases

Comparisons of the clinicopathological characteristics of the IEFN and IEIN samples are shown in Table 1. The frequency of the depressed type was significantly lower in the IEFN than IEIN cases (*p* < 0.01; Table 1). In addition, there was a significantly higher frequency of moderately differentiated tumors among the IEIN than IEFN cases. Finally, we examined the presence of mucosal atrophy and intestinal metaplasia in the mucosa surrounding the tumors. Every IEFN and IEIN case exhibited both mucosal atrophy and intestinal metaplasia, except for one IEIN case that lacked intestinal metaplasia.

### Differences in immunohistochemical marker expression between IEFN and IEIN

Although the frequency of the gastric phenotype was significantly higher in IEFN (33/42 [78.6%]) than IEIN (17/77 [22.1%]) cases (*p* < 0.001), that of the intestinal phenotype was significantly higher in the IEIN (32/77 [41.6%]) than IEFN (0/42 [0%]) cases (*p* < 0.001). There were no differences in the frequencies of the other phenotypes, i.e., mixed (IEFN versus IEIN, 9/42 [21.4%] versus 27/77 [35.1%]) and unclassified (IEFN versus IEIN, 0/42 [0%] versus 1/77 [1.3%]) phenotypes. There were no significant differences in the frequencies of CDX2 expression (IEFN versus IEIN, 25/42 [59.5%] versus 48/77 [62.3%]) or p53 overexpression (IEFN versus IEIN, 5/42 [11.9%] versus 9/77 [11.7%]) between the IEFN and IEIN cases. However, there was a significant difference in the frequency of intranuclear expression of β-catenin between the IEFN (0/42 [0%]) and IEIN (40/77 [51.9%]) cases (*p* < 0.001).

### Difference in the MSI between IEFN and IEIN

There was no statistical difference in the frequency of MSI between IEFN (1/42 [2.4%]) and IEIN (7/77 [9.1%]).

### Difference in the methylation status between IEFN and IEIN

**Fig. 3:**
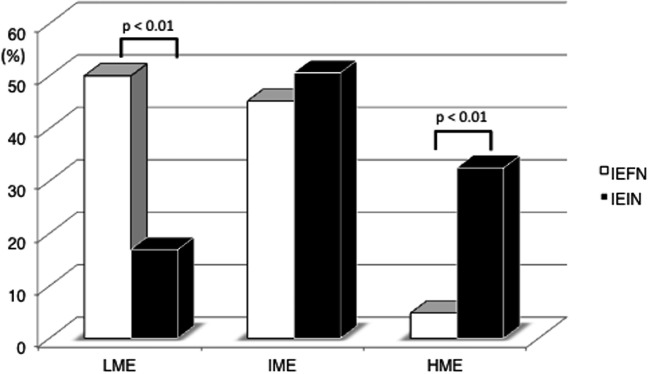
Comparison of the methylation status (LME, IME, and HME) between foveolar type neoplasia and intestinal type neoplasia. LME, low methylation epigenotype; IME, intermediate methylation epigenotype; HME, high methylation epigenotype

The frequency of LME was significantly higher in the IEFN (21/42 [50%]) than IEIN (13/77 [16.9%]) cases; however, that of HME was significantly higher in the IEIN (25/77 [32.5%]) than IEFN (2/42 [4.8%]) cases (*p* < 0.001). There were no differences in the IME frequency between IEFN (19/42 [45.2%]) and IEIN (39/77 [50.6%]). These results are shown in Fig. 3.

### Difference in the AI frequency at cancer-related chromosomal loci between IEFN and IEIN

The AI frequencies at 1p, 5q, 18q, and 22q were significantly higher in the IEFN than IEIN cases, whereas no significant differences were found in the AI frequencies at the other loci examined, including 3p, 4q, 8p, 9p, 18q, and *TP53* (Table 2).

**Table 2:** Comparison of allelic imbalance between intraepithelial foveolar type neoplasia and intraepithelial intestinal type neoplasia

	IEFN AI/IC (%)	IEIN AI/IC (%)	*p* value
Total	42	77	
1p	11/30 (36.7)	7/60 (10.7)	*p* = 0.0052
3p	3/29 (10.3)	10/58 (18.2)	*p* = 0.5950
4q	5/23 (21.7)	13/65 (19.0)	*p* = 0.8590
5q	20/34 (58.8)	21/64 (32.2)	*p* = 0.0130
8p	6/21 (28.6)	11/59 (19.6)	*p* = 0.3396
9p	4/18 (22.2)	7/57 (12.7)	*p* = 0.5110
13q	2/14 (14.3)	6/42 (15.4)	*p* = 0.6592
*TP53*	4/31 (12.9)	6/58 (10.9)	*p* = 0.9905
18q	13/35 (37.1)	10/64 (16.9)	*p* = 0.0154
22q	12/25 (49.0)	9/61 (15.5)	*p* = 0.0011

*IEFN*, intraepithelial foveolar type neoplasia; *IEIN*, intraepithelial intestinal type neoplasia; *AI*, allelic imbalance; *IC*, informative cases

### Difference in the frequency of *APC* promoter 1B mutations between IEFN and IEIN

Among the 42 tumors (20 IEFN and 22 IEIN), there were no differences in the frequencies of *APC* promoter 1B mutations between the IEFN (1/20) and IEIN (0/22) cases. The mutation observed in the one IEFN case was a codon 180–181 1-bp deletion.

## Discussion

In general, gastric intraepithelial neoplasia is histologically classified into intestinal and gastric types according to histological features [2]; the gastric type can be further divided into the foveolar and pyloric types [23]. Whereas the intestinal type can progress to intestinal type adenocarcinoma via adenoma, IEFN may occur de novo from the native gastric mucosa, leading to gastric type adenocarcinoma [24]. However, the progression of the gastric type is not clear [3, 24, 25]. Pyloric tumors resemble the pyloric gland histologically and are characterized molecularly by frequent *GNAS* mutations and a low rate of loss of heterozygosity [25]. However, the clinicopathological and molecular findings of IEFN are not fully understood. Although the histological classification system of the WHO is used worldwide, IEFN of the stomach has not been defined and is not well understood by pathologists. This is the first study to identify the detailed molecular alterations in IEFN.

Gastric adenocarcinoma and proximal polyposis of the stomach (GAPPS) is a rare hereditary GC characterized by proximal gastric polyposis and increased risk of early-onset GC. Recent studies have shown that the histological types of GC occurring in GAPPS may be both IEFN and IEIN. In addition, the specific mutations that characterize the rare histological subtype of IEFN have not yet been identified. Accordingly, gastric IEFN can be classified into two subtypes, i.e., sporadic and familial adenomatous polyposis (FAP), the latter being a histological type observed in GAPPS [26, 27]. This finding suggested that specific mutations in *APC* occurring in IEFN may be located in *APC* promoter 1B. In the current study, we attempted to examine whether mutations in the *APC* promoter 1B region were found in IEFN and IEIN. Our results showed that *APC* exon 1B mutations, a feature of FAP, were a rare mutation type in IEFN. Despite similar histological features, we therefore hypothesized that different genetic alterations existed between the sporadic and FAP subtypes, accounting for their different biological behaviors.

A recent study showed that gastric tumors exhibiting a raspberry-like appearance histologically resemble gastric IEFN and are closely associated with the absence of *Helicobacter pylori* infection [28]. These raspberry-appearing tumors are a representative tumor type originating from gastric mucosa not infected with *H. pylori* [29]. This is in contrast to the findings of the current study demonstrating that IEFN was closely related to mucosal atrophy and intestinal metaplasia. According to the histological classification, there are two subtypes of IEFN, conventional IEFN and raspberry types. Although the histological findings are similar between the two subtypes, the molecular alterations might differ [28]. Despite advances in the evaluation of GC, the molecular alterations characterizing these two subtypes of sporadic IEFN are not fully understood. In the current study, raspberry-appearing tumors were not included. We plan to identify the differences in molecular alterations between the two sporadic IEFN subtypes in the near future.

Intranuclear accumulation of β-catenin is frequently observed in GC. β-Catenin intranuclear accumulation plays a tumorigenic role by promoting tumor cell proliferation [30] and results from Wnt signaling activation, one of the most important molecular alterations in GC [30]. In the current study, no intranuclear accumulation of β-catenin was observed in the IEFN cases examined, suggesting that Wnt signaling plays a minor role in the development of IEFN. The signaling pathways that directly promote tumor progression may differ between IEIN and IEFN.

CDX2 is a transcription factor expressed in intestinal cells [28] and is a good marker of intestinal differentiation [23, 31]. CDX2 has been evaluated with regard to the intestinal phenotype [23, 31]. Therefore, the association between CDX2 expression and the mucin phenotype is important in the evaluation of GC pathogenesis. In the current study, CDX2 was highly expressed in IEFN, which was unexpected considering that IEFN was associated with the gastric phenotype. A recent study showed aberrant expression of CDX2 in not only colorectal cancer but also GC and significantly higher CDX2 expression in *H. pylori*–positive intestinal metaplasia [31]. Expression of CDX2 in IEFN may be associated with *H. pylori* infection, given that atrophic changes and intestinal metaplasia are frequently found in this lesion type. This finding suggests that IEFN in the current study may be different from lesions characterized by non-intestinal metaplastic mucosa.

Recent studies have shown that DNA methylation plays an important role in gastric carcinogenesis [32]. Numerous studies have implicated aberrant DNA methylation at numerous gene loci in different human samples and models of gastric tumorigenesis [33]. In the current study, we found that high-to-intermediate levels of DNA methylation were more common in IEIN than in IEFN. Cancer-induced methylation changes in cancer-related genes have potential pathological implications in terms of early tumorigenesis [32, 33]. However, our current findings suggested that DNA methylation may play a minor role in the early development of IEFN compared with IEIN. Although atrophic gastritis and intestinal metaplasia, which are expected to exhibit high DNA methylation levels, are frequently found in IEFN [32, 33], the IEFN cases demonstrated an LME in the current study. This suggested that the pathogenesis of DNA methylation may differ between gastric IEIN and IEFN.

AI is a genomic change representing genomic instability [34]. AI is also thought to be an aggressive factor correlated with the tumor grade in neoplastic conditions [10]. In the current study, AIs at 1p, 5q, 18q, and 22q were frequently found in the IEFN compared with the IEIN cases. These findings suggested that despite the low-grade nature of the lesion, IEFN demonstrating AIs at multiple foci, such as 1p, 5q 18q, and 22q, may have the risk of progressing to severe dysplasia, dedifferentiated lesions, or more advanced disease. Due to the low grade of IEFN, patients with this disease may not receive aggressive treatment or monitoring, despite the presence of multiple AIs predicting tumor aggressiveness [9, 10]. This finding may have clear implications for the treatment of IEFN, although the recommended frequency of follow-up remains to be determined. It is unclear whether high-risk lesions with multiple AIs should be monitored aggressively for clinical progression. We suggest that this type of lesion, appearing initially to be histologically indolent, is pathologically important because multiple AIs may be involved.

Gastric hyperplastic polyps (GHPs) are the most common type of polyps occurring in the stomach [35]. GHPs are considered benign, and they rarely progress to dysplasia or adenocarcinoma [35]. Although GHP resembles IEFN histologically, GHP differs from IEFN in terms of molecular alterations (as shown in the current study) and clinical treatment [35]. However, the differential diagnosis of GHP and IEFN may be difficult for general pathologists. If IEFN is left untreated, it will progress to a more malignant stage (e.g., submucosal invasion). In contrast, untreated GHP may not progress to a more malignant stage. GHP itself is considered a stable disease according to mutation analyses using next-generation sequencing [35]. Pathologists should be careful not to confuse GHP with IEFN histologically.

There are some limitations to the current study. First, a limited number of genetic markers of AI to identify carcinogenesis of IEFN were evaluated. A recent study showed that genome-wide analyses, such as those using The Cancer Genome Atlas, are preferential for examining genomic changes in human neoplasia [8]. However, such comprehensive analyses may not be suitable for paraffin-embedded tissue samples. PCR-based analyses, including AI analyses, are effective for examining paraffin-embedded tissues. Second, we did not have a validation cohort for molecular analysis of IEFN, given that this lesion is relatively rare. Additional studies investigating the molecular alterations involved in IEFN will be needed in the near future.

In conclusion, no β-catenin intranuclear accumulation was observed in IEFN lesions, suggesting that, unlike IEIN, Wnt signaling was not activated in IEFN. In addition, the IEFN cases were characterized by AIs at multiple foci, including 1p, 5q, and 22q, which was a good indicator of genomic instability. Our results suggested that IEFN acquired more aggressive behaviors than IEIN. In addition, this lesion may be overlooked as a candidate for endoscopic treatment. The pathological and molecular alterations in IEFN will need to be evaluated in greater detail in the near future.

## Electronic supplementary material

Supplementary Figure 1. a. MUC2, b. MUC2, CD10, c. MUC6, d. CD10. The cut-off value was set at a score of 2 (> 10%). (PNG 543 kb)

High resolution image (TIFF 1142 kb)

Supplementary Figure 2. a. CDX2, b. p53, c. β-catenin. The cut-off value was set at a score of 2 (> 10%). (PNG 456 kb)

High resolution image (TIFF 1142 kb)

ESM 1 (DOCX 12 kb)

ESM 2 (DOCX 19 kb)
