# Supplementary figures and images for: The clinicopathological and molecular features of sporadic gastric foveolar type neoplasia
Source: Virchows Arch. 2020 Jun 12;477(6):835–44. doi: 10.1007/s00428-020-02846-0 (PMC7683467; doi:10.1007/s00428-020-02846-0)

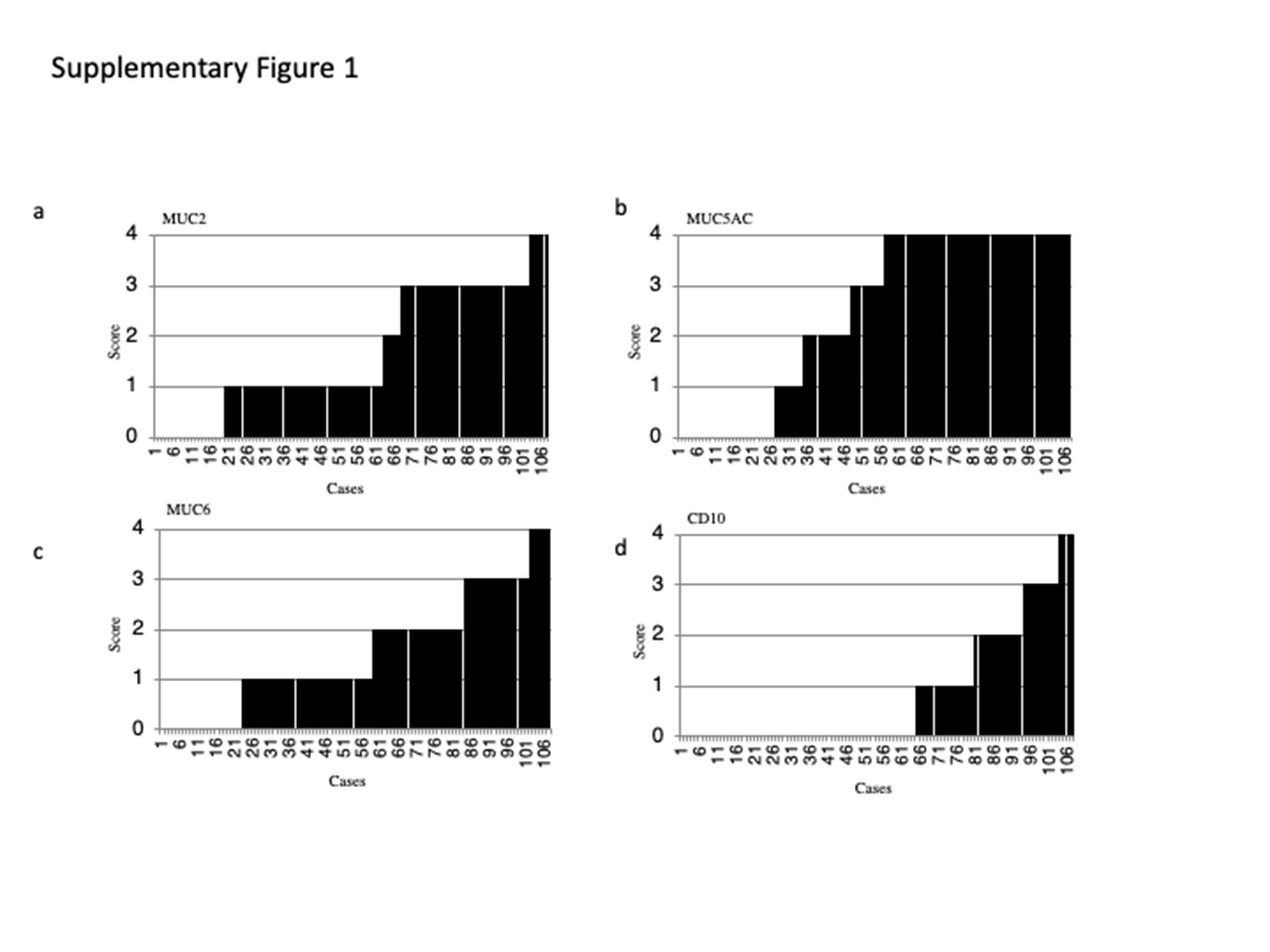

Supplement: Supplementary file 1 — a. MUC2, b. MUC2, CD10, c. MUC6, d. CD10. The cut-off value was set at a score of 2 (> 10%). (PNG 543 kb) [file 428_2020_2846_Fig4_ESM.png]

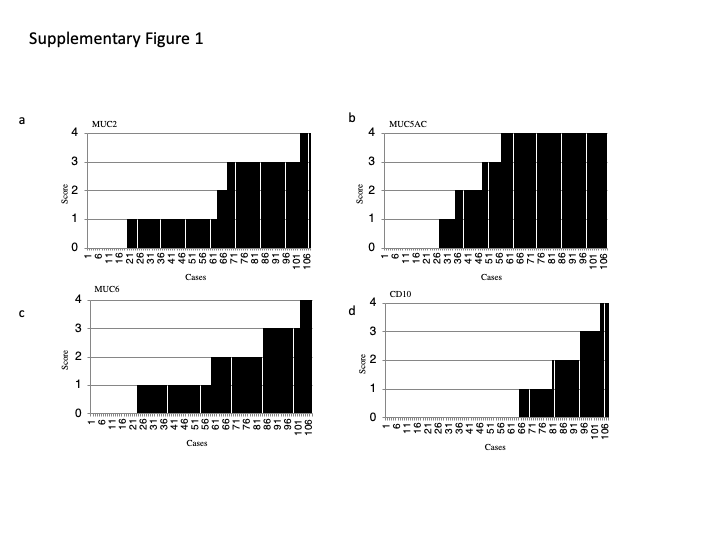

Supplement: Supplementary file 2 — High resolution image (TIFF 1142 kb) [file 428_2020_2846_MOESM1_ESM.tiff]

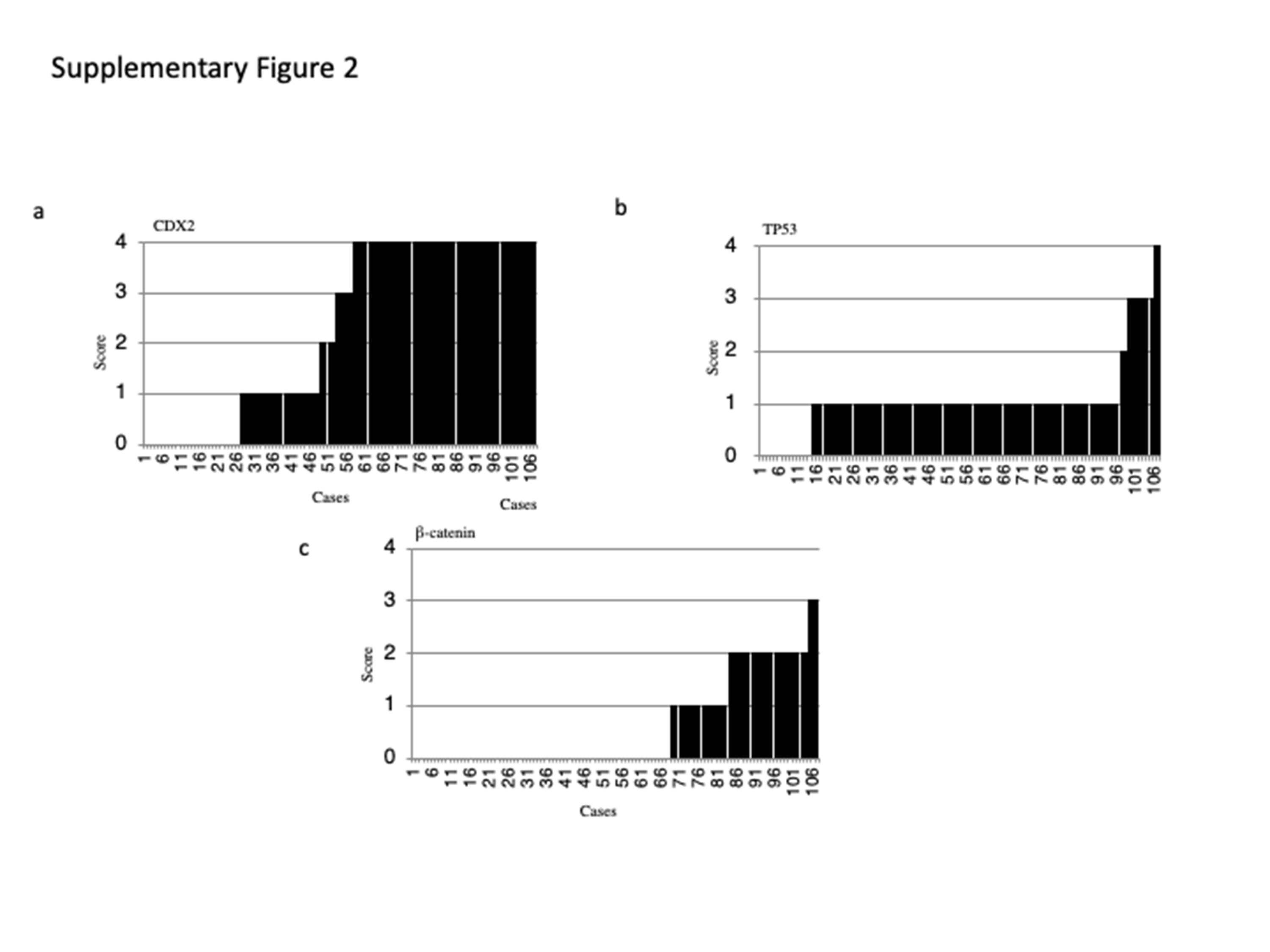

Supplement: Supplementary file 3 — a. CDX2, b. p53, c. β-catenin. The cut-off value was set at a score of 2 (> 10%). (PNG 456 kb) [file 428_2020_2846_Fig5_ESM.png]

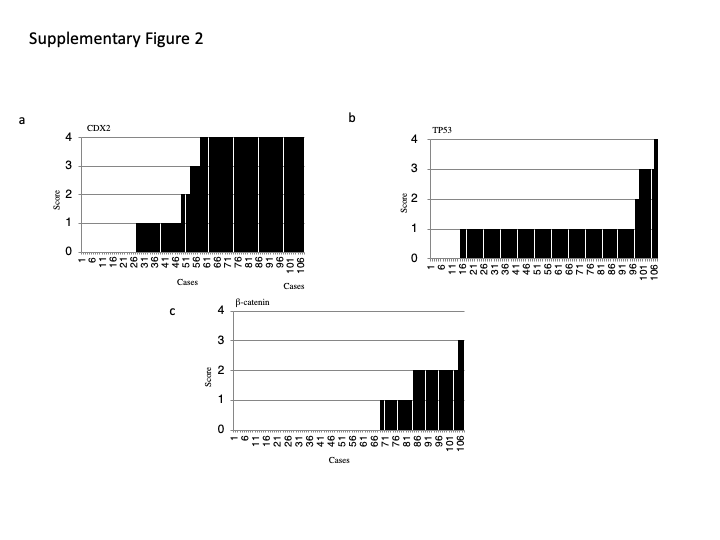

Supplement: Supplementary file 4 — High resolution image (TIFF 1142 kb) [file 428_2020_2846_MOESM2_ESM.tiff]
