# Supplementary material for: The clinicopathological and molecular features of sporadic gastric foveolar type neoplasia
Source: Virchows Arch. 2020 Jun 12;477(6):835–44. doi: 10.1007/s00428-020-02846-0 (PMC7683467; doi:10.1007/s00428-020-02846-0)
Supplement: Supplementary file 5 — (DOCX 12 kb) [file 428_2020_2846_MOESM3_ESM.docx]

Supplementary Table: The primer sequence of APC promoter 1B for nested PCR

| Primer | Sequence |
| --- | --- |
| 1st forward | 5'-TGGGAACAGCATCGAGCCAA-3' |
| 1st reverse | 5'-ACCCATTGCGCCTGCGCATA-3' |
| 2nd forward | 5'-AGCAGCGGCTAGGCTTCCG-3' |
| 2nd reverse | 5'-ATAACAGGCTCTAGTCTCCG-3' |
