# Supplementary material for: The clinicopathological and molecular features of sporadic gastric foveolar type neoplasia
Source: Virchows Arch. 2020 Jun 12;477(6):835–44. doi: 10.1007/s00428-020-02846-0 (PMC7683467; doi:10.1007/s00428-020-02846-0)
Supplement: Supplementary file 6 — (DOCX 19 kb) [file 428_2020_2846_MOESM4_ESM.docx]

Supplementary Table 1 for reviewer only: Clinicopathological findings of intraepithelial foveolar type neoplasia, intraepithelial intestinal type neoplasia with low grade dysplasia and intraepithelial intestinal type with high grade dysplasia

|  |  | IEFN (%) | IEINLD (%) | IEINHD (%) | *P*-value |
| --- | --- | --- | --- | --- | --- |
| Total |  | 42 | 77 | 58 |  |
| Sex | Man : Woman | 28 : 14 | 58 : 19 | 42 : 16 | N.S. |
| Age (year) | Range (median) | 25-87 (71) | 54-87 (72) | 49-84 (71) | N.S. |
| Size (mm) | Range (median) | 4-53 (15) | 10-103 (19) | 4-108 (20) | N.S. |
| Locus | Upper | 8 (19.0) | 14 (18.1) | 5 (8.6) |  |
|  | Middle | 13 (31.0) | 33 (42.9) | 25 (43.1) |  |
|  | Lower | 21 (50) | 30 (39.0) | 28 (48.3) | N.S. |
| Macroscopic type | Protruded type | 7 (16.7) | 2 (2.6) | 7 (12.1) |  |
|  | Flat elevated type | 27 (64.3)* | 40 (51.9) | 20 (34.5)* |  |
|  | Flat type | 1 (2.4) | 7 (9.1) | 2 (3.4) |  |
|  | Depressed type | 7 (16.7)* | 28 (36.4) | 29 (50.0)* | P<0.001 |
| Mucosal atrophy | Negative | 0 (0) | 0 (0) | 0 (0) |  |
|  | Positive | 42 (100) | 77 (100) | 58 (100) | N. S. |
| Intestinal metaplasia | Negative | 0 (0) | 1 (1.3) | 1 (1.7) |  |
|  | Positive | 42 (100) | 76 (98.7) | 57 (98.3) | N.S. |

*, p<0.01

IEFN, intraepithelial foveolar type neoplasia; IEINLG, intraepithelial intestinal type neoplasia with low grade dysplasia;

IEINHD, intraepithelial conventional type with high-grade dysplasia; N.S., not significant

Supplementary Table 2 for reviewer only: Comparison of allelic imbalance between intraepithelial foveolar type neoplasia, intraepithelial intestinal type neoplasia with low grade dysplasia and intraepithelial intestinal type neoplasia with high grade dysplasia

|  | IEFN AI/IF (%) | IEINLG AI/IF (%) | IEINHD AI/IF (%) | *P*-value |
| --- | --- | --- | --- | --- |
| Total | 42 | 77 | 58 |  |
| 1p | 11/30 (36.7)** | 7/60 (10.7)** | 8/36 (22.2) | p=0.0211 |
| 3p | 3/29 (10.3) | 10/58 (18.2) | 13/40 (32.5) | p=0.0632 |
| 4q | 5/23 (21.7) | 13/65 (19.0) | 10/41 (24.4) | p=0.8671 |
| 5q | 20/34 (58.8)** | 21/64 (32.2)** | 19/46 (41.3) | p=0.0454 |
| 8p | 6/21 (28.6) | 11/59 (19.6) | 8/44 (18.2) | p=0.5729 |
| 9p | 4/18 (22.2) | 7/57 (12.7) | 13/43 (30.2) | p=0.0916 |
| 13q | 2/14 (14.3) | 6/42 (15.4) | 8/33 (24.2) | p=0.5250 |
| TP53 | 4/31 (12.9) | 6/58 (10.9)* | 16/42 (38.1)* | p=0.00253 |
| 18q | 13/35 (37.1)** | 10/64 (16.9)** | 10/46 (21.7) | p=0.04980 |
| 22q | 12/25 (49.0)* | 9/61 (15.5)* | 12/43 (27.9) | p=0.0053 |

*, p<0.01; **, p<0.01

IEFN, intraepithelial foveolar type neoplasia; IEINLG, intraepithelial intestinal type neoplasia with low grade dysplasia;

IEINHD, intraepithelial conventional type with high-grade dysplasia; AI, allelic imbalance; IC, informative case
